# Supplementary material for: Physiological Features of the Neural Stem Cells Obtained from an Animal Model of Spinal Muscular Atrophy and Their Response to Antioxidant Curcumin
Source: Int J Mol Sci. 2024 Jul 31;25(15):8364. doi: 10.3390/ijms25158364 (PMC11313061; doi:10.3390/ijms25158364)
Supplement: Supplementary file 1 [file ijms-25-08364-s001.zip › Supp Fig Tiff/Adami et al 2024 Supplemental Figures.pdf]

Supplemental Figures

Fig. 1 sup

Representative growth curve graph.

A comparison of proliferation capability between WT and SMA-ctr and -Cur treated samples. The experiment was performed on samples WT7 and SMA7 (derived from the same nest). Blu: WT, green SMA, light colors: ctr; dark colors: Cur. Data was collected from 50 days of growth. Dashed lines represent the linear regression. Each node of the curve represents an *in vitro* passage by means of mechanical dissociation.

Fig. 2 sup

Growth curve slopes

This figure represents the comparison between the slopes of the growth curves of WT and SMA samples (treated or not treated with Cur). White: WT-ctr; white pattern WT-Cur; green: SMA-ctr; green pattern: SMA-Cur. * vs. WT-ctr, $^{\$}$ vs. SMA-ctr, ° vs. WT-Cur. No significant differences were detected in the comparison between WT-ctr and SMA-Cur. $^{*,\circ}$ $p<0.05$; $^{**,\$\$}$ $p<0.01$. Statistical analysis was performed using the two-tailed paired t-test.

Fig. 3 sup

Proliferation doubling times comparison between WT and SMA samples treated or not treated with Cur.

The average time (in days) required to double the cell population was calculated with non-linear regression analysis. White: WT-ctr; white pattern WT-Cur; green: SMA-ctr; green pattern: SMA-Cur. * vs. WT-ctr, $^{\$}$ vs. SMA-ctr. $^{*,\$}$ $p<0.05$. No significant differences were detected in the comparison between WT-ctr and SMA-Cur. Statistical analysis was performed using the one-tailed paired t-test

Fig. 4 sup.

Clonogenic capability study

The clonal ability of NSCs obtained from WT and SMA mice, treated or not treated with Cur was assessed by a clonal assay at the start and at the end (50 days) of the proliferation (growth curve) analysis. White: WT-ctr; white pattern WT-Cur; green: SMA-ctr; green pattern: SMA-Cur; sky blue: WT-ctr 50 days; sky blue pattern: WT-Cur 50 days; light green: SMA-ctr 50 days; light green pattern: SMA-Cur 50 days. * vs. WT-ctr, ° vs. SMA-ctr, $^{\$}$ vs. WT-ctr 50 days, $^{\#}$ vs. SMA-ctr 50 days, $^{\&}$ vs. WT-Cur 50 days. *$p<0.05$; $^{**,\$\$}$ $p<0.01$; $^{\circ\circ\circ,\$\$\$,\#\#\#,\&\&\&}$$p<0.001$. No significant differences were detected in the comparison between WT-ctr and SMA-Cur at the start and at the end of the proliferation analysis. No significant differences were detected in the comparison between WT-Cur and SMA-Cur at the start of the proliferation analysis. Statistical analysis was performed using the two-tailed paired t-test.

Fig. 5 sup

Stemness markers expression.

Nestin and SOX2 were analyzed through immunocytochemistry.

A) representative images. B) Primary antibody omission for the same samples presented in B. Red: Nestin staining, green: SOX2 staining; blue:

DAPI staining; scale bar 20 μm. Statistical analysis was performed using the two-tailed paired t-test.

Fig. 6 sup.

Metabolic activity study

An MTT assay was used to study the metabolic activity of WT and SMA treated or not treated with Cur. Cell viability is expressed in arbitrary units after three days of *in vitro* culture. White: WT-ctr; white pattern WT-Cur; green: SMA-ctr; green pattern: SMA-Cur. $^{\$}$ p =0.0272.
No differences were detected among the other samples and treatments. Statistical analysis was performed using the two-tailed paired t-test.

Fig. 7 sup

Evaluation of SMN protein levels in WT and SMA samples treated or not treated with Cur. Three WT protein samples and three or four SMA samples, treated or not treated with Cur, were separated through electrophoresis and transferred to a PVDF membrane. Total protein per lane was measured (as a normalization factor), and SMN protein levels were detected using NB100-1936 Novus Biologicals antibody. A: White: WT-ctr; white pattern WT-Cur; green: SMA-ctr; green pattern: SMA-Cur. * vs. WT-ctr; $^{\$}$ vs. SMA-ctr. *** $p<0.001$, \$ $p<0.05$. B: SYPRO Ruby Protein Blot Stain; C: Immunoblot. The size of the molecular weight (MW) markers is displayed on the left; the position of the SMN proteins is indicated on the right, and red represents the MW of SMN proteins. Statistical analysis was performed using the one-tailed paired t-test.

Fig. 8 sup

NRF2 nuclear/cytoplasmic ratio

We measured the ratio of nuclear and cytoplasmic NRF2 through immunocytochemical analysis. A) Assessment of NRF2 distribution. White: WT-ctr, white pattern WT-Cur, green: SMA-ctr; green pattern: SMA-Cur. * vs. WT-ctr; $^{\$}$ vs. SMA-ctr. **** $p<0.0001$, $^{\$}$ $p<0.05$. B) Representative images used for the analysis. Inserts are enlarged 3X. C) Primary antibody omission for the samples presented in B. Green: NRF2 staining; blue: DAPI staining; scale bar 100 μm. Statistical analysis was performed using the one-tailed paired t-test.

Fig. 9 sup

Curcumin dose response

The effect of Cur was evaluated in terms of proliferation effects. Different concentrations were used to treat WT-NSCs: white: no Cur, white vertical pattern: (vehicle, DMSO 1:1000); 0.1 μM Cur: ●; 0.3 μM Cur: ●; 0.5 μM Cur: ●; 1 μM Cur: ●; 10 μM Cur: ●; 25 μM Cur: ●; and 50 μM Cur: ●. * vs. CTR. **$p<0.01$; ***$p<0.001$; ****$p<0.0001$. The analysis was performed using one-way Anova
