# Supplementary figures and images for: Physiological Features of the Neural Stem Cells Obtained from an Animal Model of Spinal Muscular Atrophy and Their Response to Antioxidant Curcumin
Source: Int J Mol Sci. 2024 Jul 31;25(15):8364. doi: 10.3390/ijms25158364 (PMC11313061; doi:10.3390/ijms25158364)

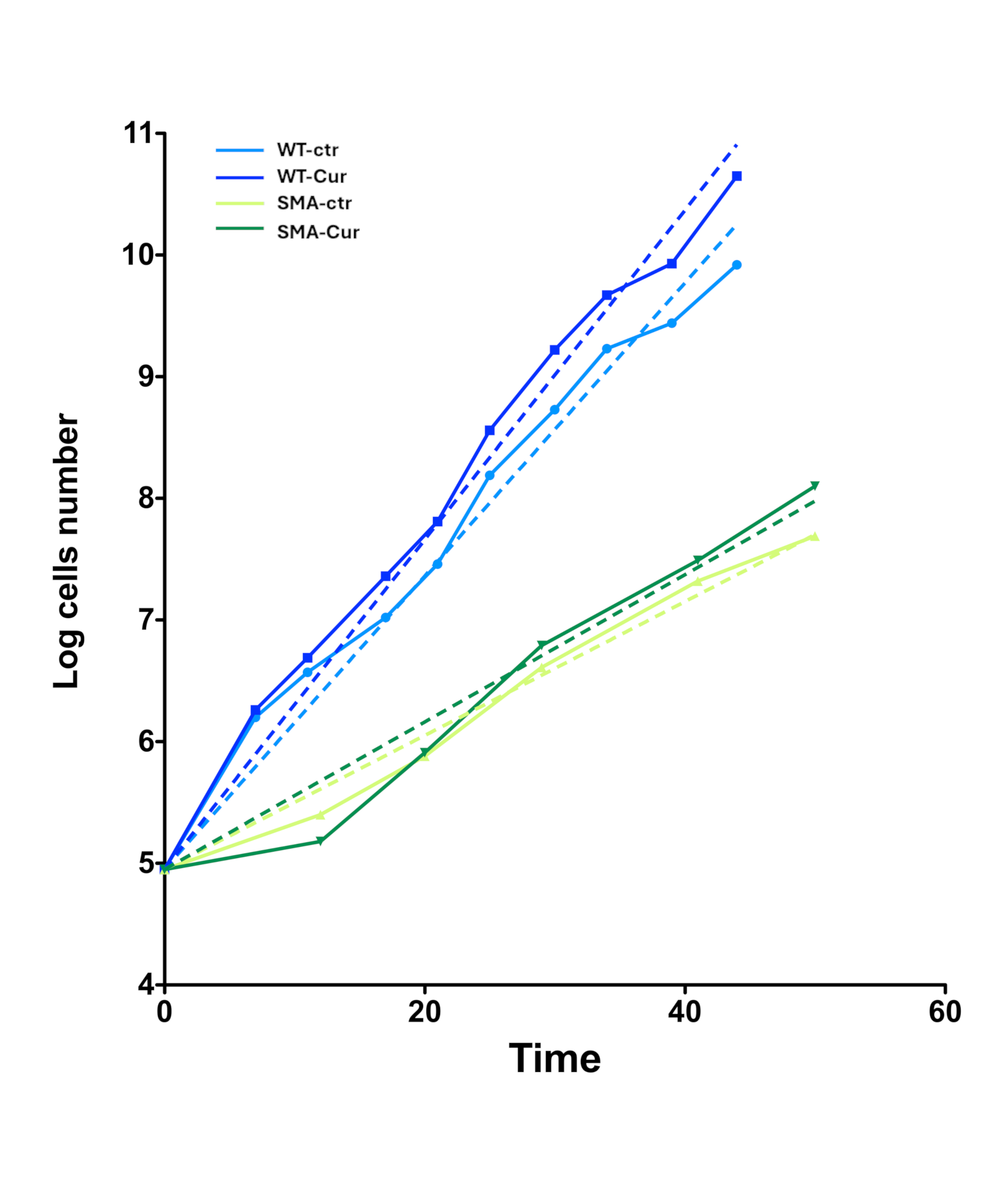

Supplement: Supplementary file 1 [file ijms-25-08364-s001.zip › Supp Fig Tiff/Fig 1 sup.tif]

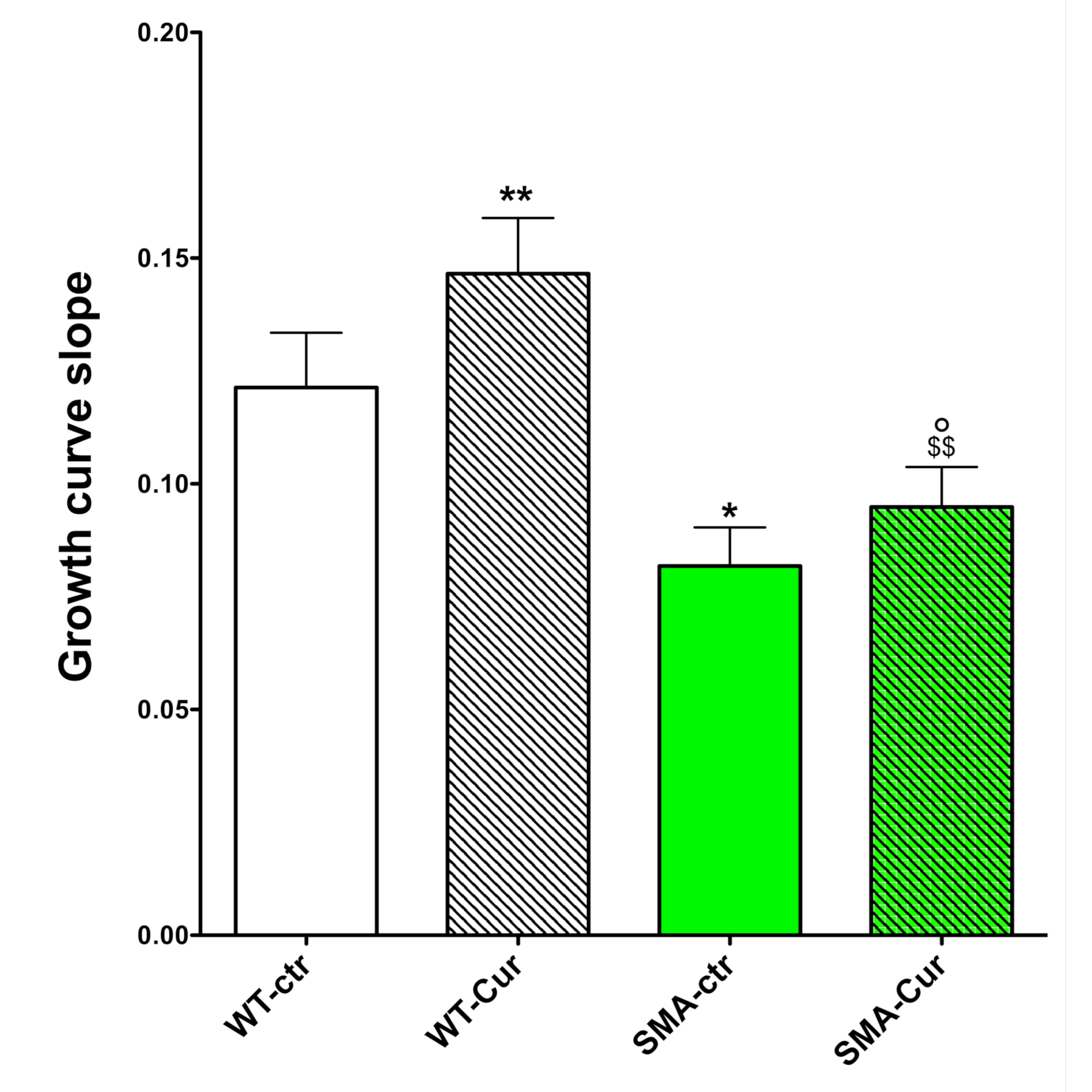

Supplement: Supplementary file 1 [file ijms-25-08364-s001.zip › Supp Fig Tiff/Fig 2 sup.tif]

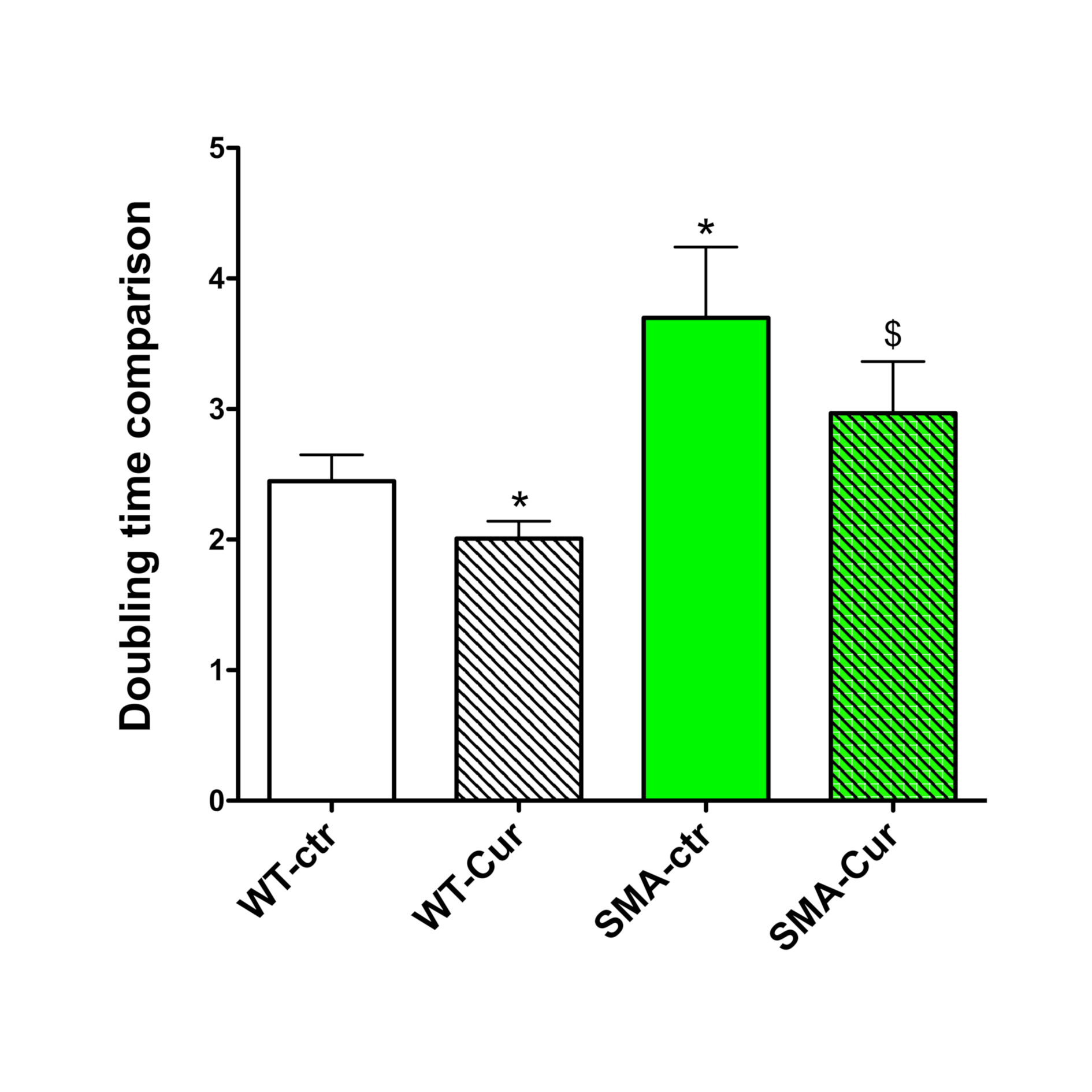

Supplement: Supplementary file 1 [file ijms-25-08364-s001.zip › Supp Fig Tiff/Fig 3 sup.tif]

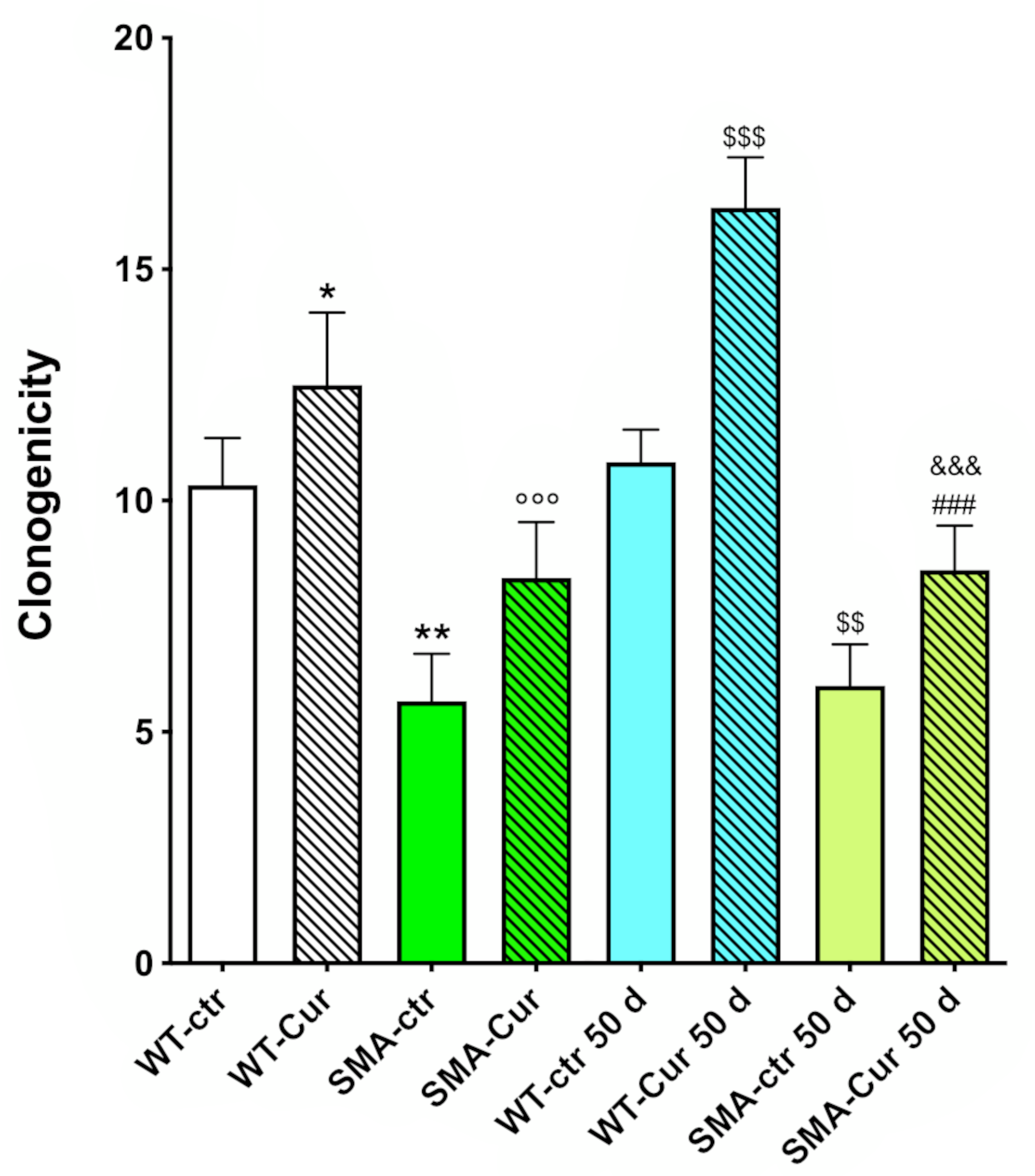

Supplement: Supplementary file 1 [file ijms-25-08364-s001.zip › Supp Fig Tiff/Fig 4 sup.tif]

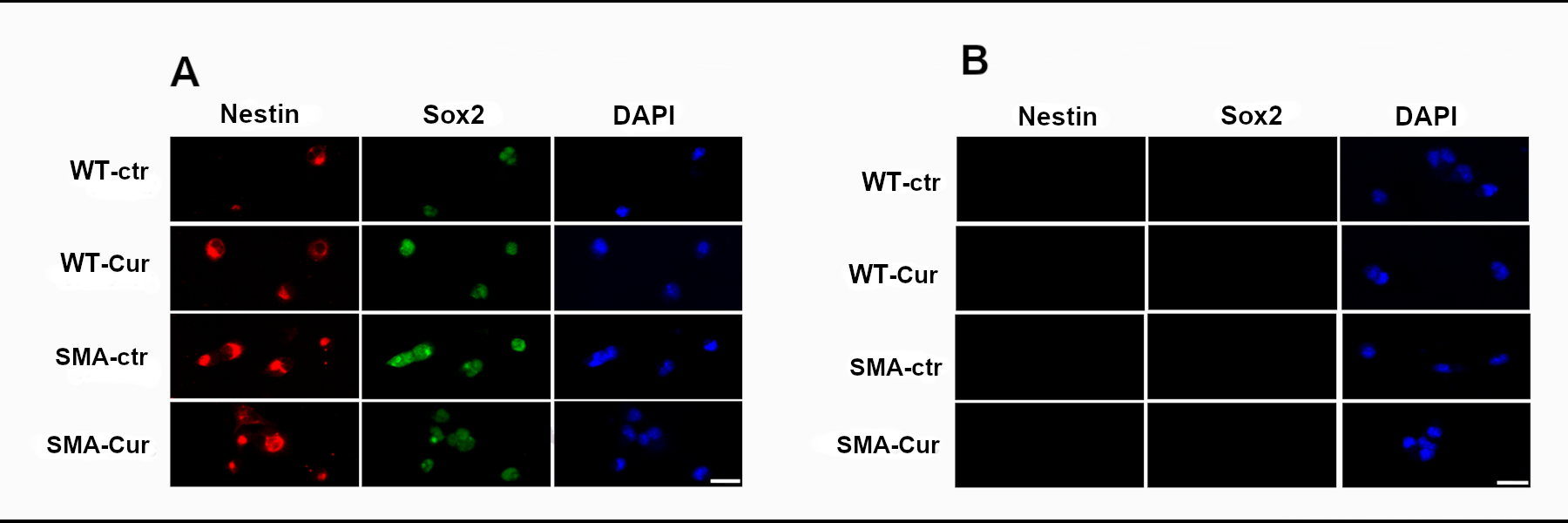

Supplement: Supplementary file 1 [file ijms-25-08364-s001.zip › Supp Fig Tiff/Fig 5 sup.tif]

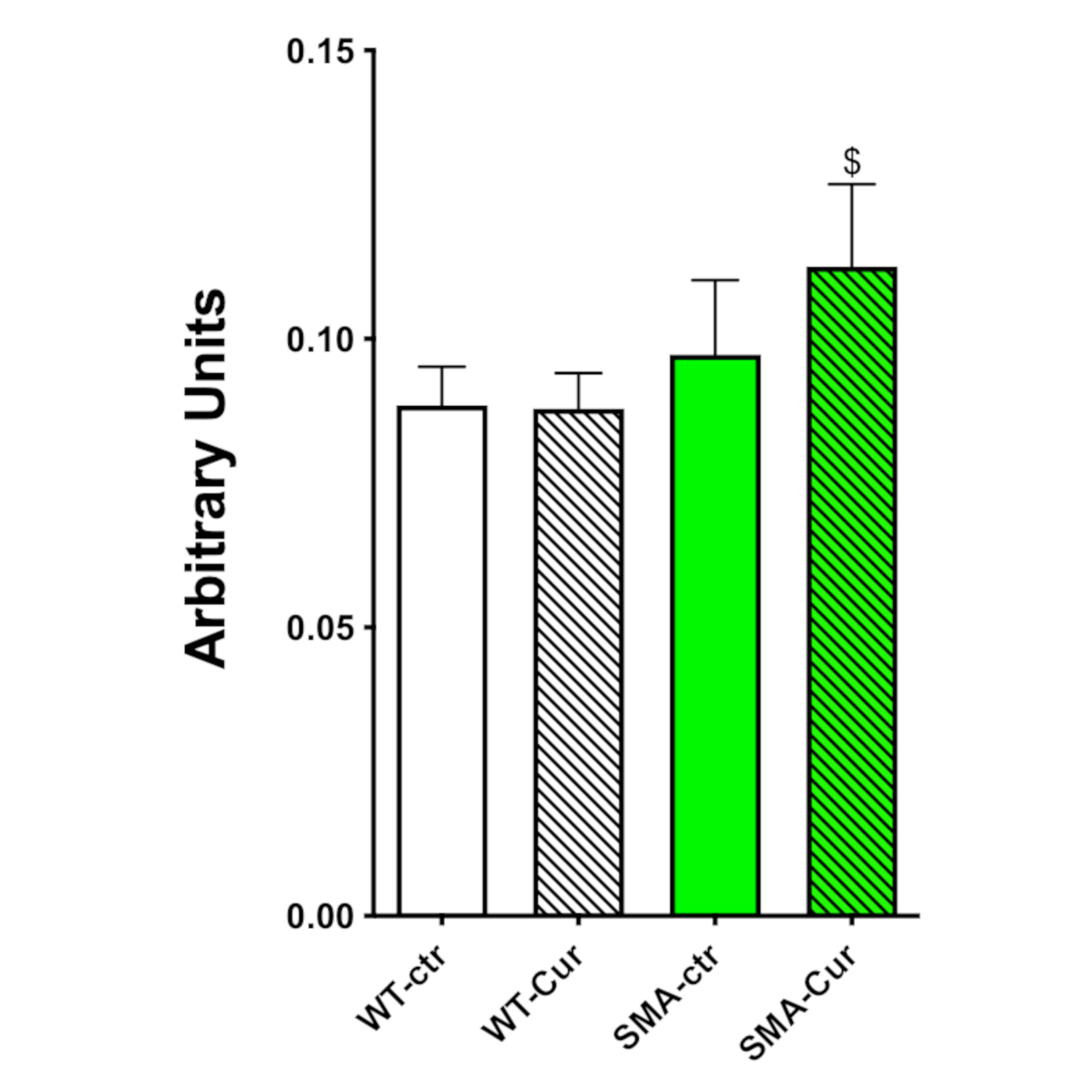

Supplement: Supplementary file 1 [file ijms-25-08364-s001.zip › Supp Fig Tiff/Fig 6 sup.tif]

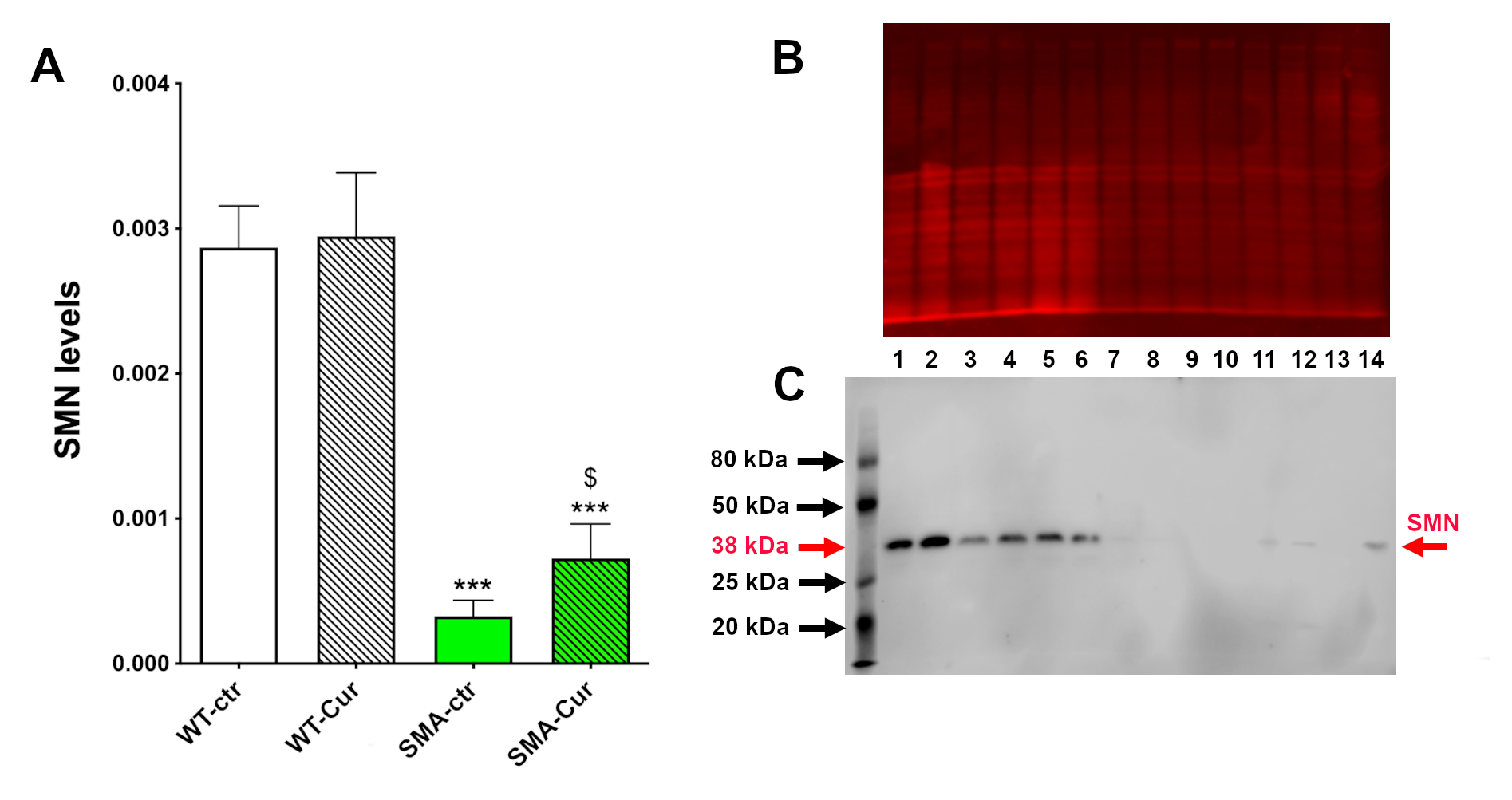

Supplement: Supplementary file 1 [file ijms-25-08364-s001.zip › Supp Fig Tiff/Fig 7 sup.tif]

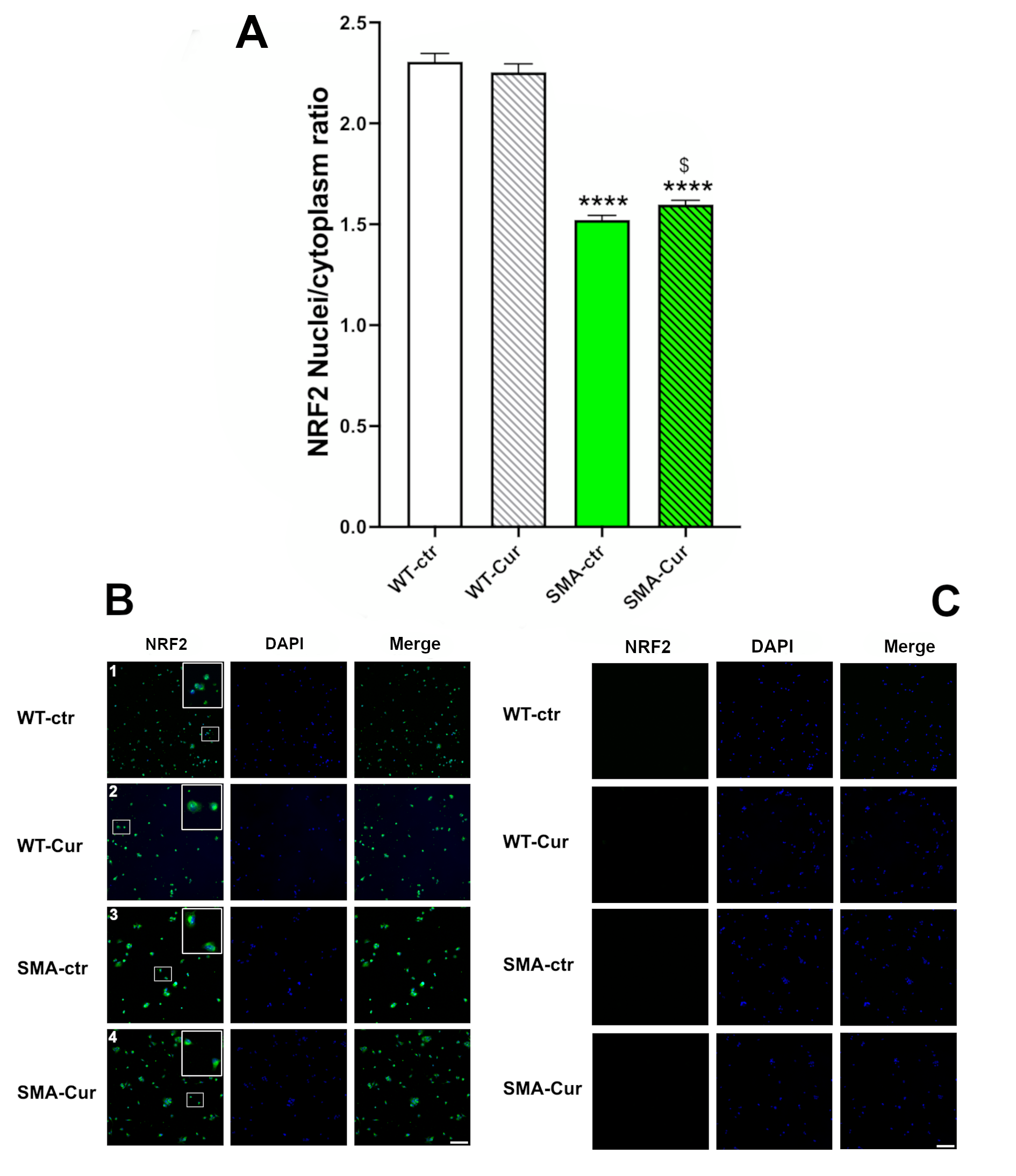

Supplement: Supplementary file 1 [file ijms-25-08364-s001.zip › Supp Fig Tiff/Fig 8 sup .tif]

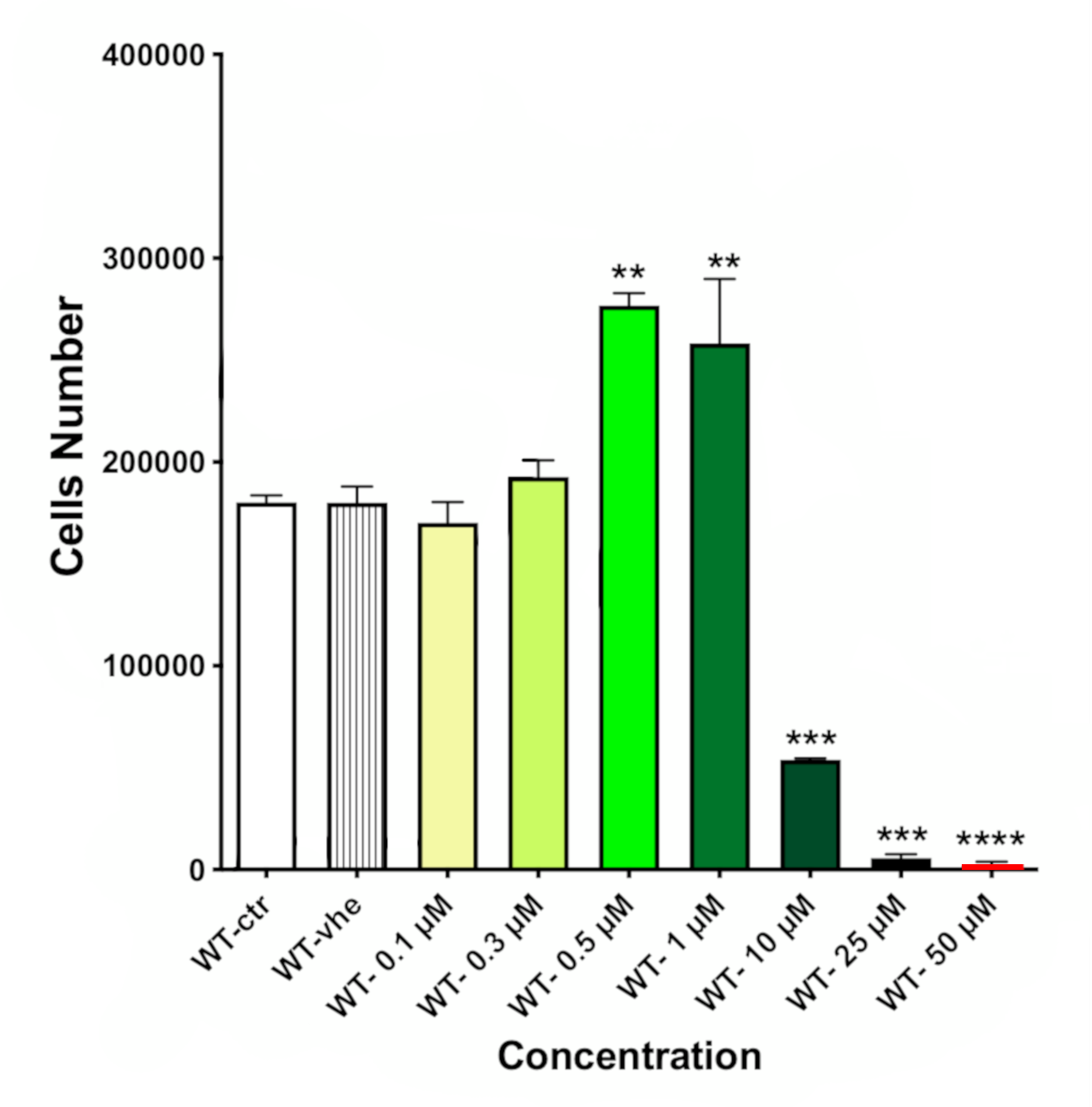

Supplement: Supplementary file 1 [file ijms-25-08364-s001.zip › Supp Fig Tiff/Fig 9 sup.tif]
